# Quantitative Analysis of Enhancement Intensity and Patterns on Contrast-enhanced Spectral Mammography

**DOI:** 10.1038/s41598-020-66501-z

**Published:** 2020-06-17

**Authors:** Ying Liu, Shuang Zhao, Juan Huang, Xueqin Zhang, Yun Qin, Huanhuan Zhong, Jianqun Yu

**Affiliations:** Department of Radiology, Sichuan University West China Hospital, No. 37 Guoxue Alley, Chengdu, 610041 Sichuan Province China

**Keywords:** Breast cancer, Pathology

## Abstract

CESM is an emerging digital mammography technology with a high breast cancer detection and a limited diagnostic specificity. In order to improve specificity, we quantitatively assessed enhancement intensity of breast lesions with different pathological types and hormonal receptor status and evaluated the consistency of enhancement patterns between CESM and DCE-MRI. A total of 145 lesions were enrolled, consisting of 43 malignant (17 non-infiltrating cancers and 26 infiltrating cancers) and 99 benign lesions. The diagnostic performance of enhancement intensity in the former positions was significantly higher than that in the latter positions (AUC: 0.834 vs. 0.755, *p* = 0.0008). Infiltrating cancers showed the highest enhancement intensity, while benign lesions the lowest (mean CNR_1_: 7.6% vs. 2.7%; median CNR_1_: 6.8% vs. 2.7%). Enhancement intensity of ER or PR positive group was weaker than negative group, while HER-2 positive group was stronger than negative group. 28 patients with 28 lesions performed both CESM and DCE-MRI examinations, showing a coincidence rate of 64.2% and moderate agreement (k = 0.515) between CESM and DCE-MRI. In conclusion, quantitative analysis of enhancement characteristics is feasible to the diagnosis practice on CESM.

## Introduction

Breast cancer is the most common malignant tumor in women around the world^1^. Although mammography is generally considered the preferred screening method for breast cancer and can substantially reduce breast cancer mortality, its diagnostic performance is affected by mammary gland density and the sensitivity decreases significantly in dense breasts^2^.

Contrast-enhanced spectral mammography (CESM), an emerging digital mammography technology, has been approved by the U.S. FDA and put into clinical use in 2011. After intravenous iodine contrast agent, pairs of low-energy (LE) and high-energy images are acquired to generate the subtraction images. LE and subtraction images are applied to the diagnostic procedure. LE images are similar to full-field digital mammography (FFDM)^3^, which can clearly demonstrate the morphological features of breast lesions. On the basis of angiogenesis, breast lesions reveal enhancement and are highlighted on subtraction images by eliminating overlapping of breast fibroglandular tissues^4–6^. Accordingly, CESM dramatically improves the cancer detection and decreases the misdiagnosis rate, especially in dense breasts^7–10^.

Given that a number of benign lesions can reveal enhancement on CESM^8,11^, researchers have focused on how to differentiate breast cancers from benign lesions and improve the diagnostic specificity. Previous studies^12–14^ have proved the diagnostic performance of enhancement intensity for breast cancers and concluded that enhancement intensity of malignancies was usually stronger than that of benign lesions. Moreover, invasive cancers were inclined to enhance stronger than non-invasive cancers and benign lesions^12,14^. However, there is no research before investigating the correlation between enhancement intensity and hormonal receptor status of breast cancers. As for enhancement patterns, several published papers^4,13,15^ claimed that the distribution of enhancement patterns between benign and malignant lesions was different, which was similar to time-intensity curve (TIC) on breast dynamic contrast-enhanced magnetic resonance imaging (DCE-MRI). Whereas, it is uncertain that how is the agreement between two modalities. This study was aim to quantitatively assess enhancement intensity of breast lesions with different pathological types and hormonal receptor status on CESM and evaluate the consistency of enhancement patterns between CESM and DCE-MRI.

## Methods

### Patients

The ethnical committee of West China Hospital approved this retrospective study with a waiver of informed consent (20/08/2019, NO. 737 2019). All methods were performed in accordance with the relevant guidelines and regulations. CESM was performed on patients with clinically or ultrasonically suspected breast lesions from July 2017 to April 2019. Criteria for selecting the subjects were as follows: more than 18 years old; obtained pathological diagnosis after CESM and DCE-MRI examinations. Samples were excluded from the research if they were: pregnant or breastfeeding; had contraindications to contrast agent, such as renal dysfunction or contrast agent allergy; had a history of hormone treatment or radiation therapy or neoadjuvant chemotherapy for breast lesions before CESM examination. All patients were followed up for more than 6 months.

### CESM

CESM was performed using a GE healthcare equipment (SenoBright®). Detailed procedures have been provided elsewhere^16^. After intravenous iodine contrast agent (300 mg iodine/ml, 1.5 ml/kg body weight, flow rate 3 ml/s) for about two minutes, a series of consecutive images were acquired including standard craniocaudal (CC) and mediolateral oblique (MLO) views with breast compressed. The process of image acquisition was carried out in five minutes. For one projective position, a pair of low- and high-energy exposures was performed within 1.5 s and a subtraction image was obtained using a special algorithm. For bilateral breasts in two separate views, LE images (similar to FFDM) and subtraction images (which can reveal enhanced area clearly), can be utilized for diagnosis on the workstation.

**Figure 1:**
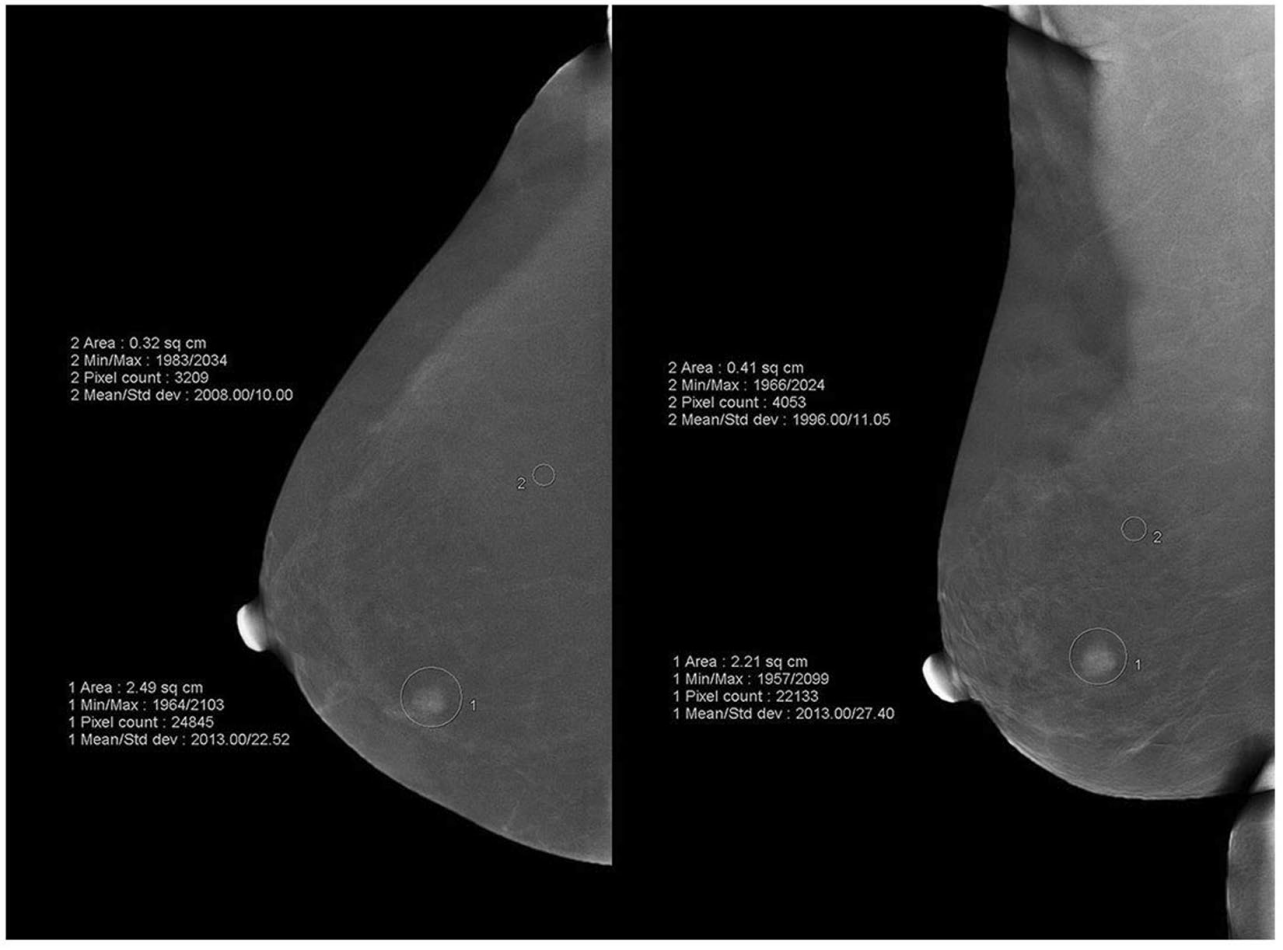
Illustration of quantitative measurements of breast lesions and background. ROIs of breast lesions should cover the entire enhanced areas and ROIs of background were as close as possible to the fatty background components of the breast and away from the enhanced lesion or enhanced breast parenchyma tissues.

On subtraction images, a positive finding referred to an enhancement region stronger than background parenchyma enhancement. A region of interest (ROI) was drawn artificially by one radiologist, and then contrast-to-noise ratio (CNR) was measured to assess enhancement intensity and relative signal difference (RSD) was calculated to value enhancement pattern. ROIs of breast lesions should cover the entire enhanced areas and ROIs of background were as close as possible to the fatty background components of the breast and away from the enhanced lesion or enhanced breast parenchyma tissues (Fig. 1). CNR reflected the signal difference between the enhanced lesion and background. CNR values of one index lesion in two projective views were calculated respectively according to time sequence. We defined CNR_1_ as the enhancement intensity in the former position, which is a relatively early phase. And CNR_2_ represented the enhancement intensity in the latter position, which is a relatively late phase. The specific calculation formulas are listed as follows:

$${\rm{CNR}}=\frac{{\rm{Sa}}-{\rm{Sb}}}{{\rm{Sb}}}$$

Sa: maximum pixel value in ROI of breast lesion

Sb: mean pixel value signal in ROI of background

$${\rm{RSD}}=\frac{{\rm{CNR}}2-{\rm{CNR}}1}{{\rm{CNR}}1}\times 100 \% $$

CNR_1_: CNR value in the former projection

CNR_2_: CNR value in the latter projection

**Figure 2:**
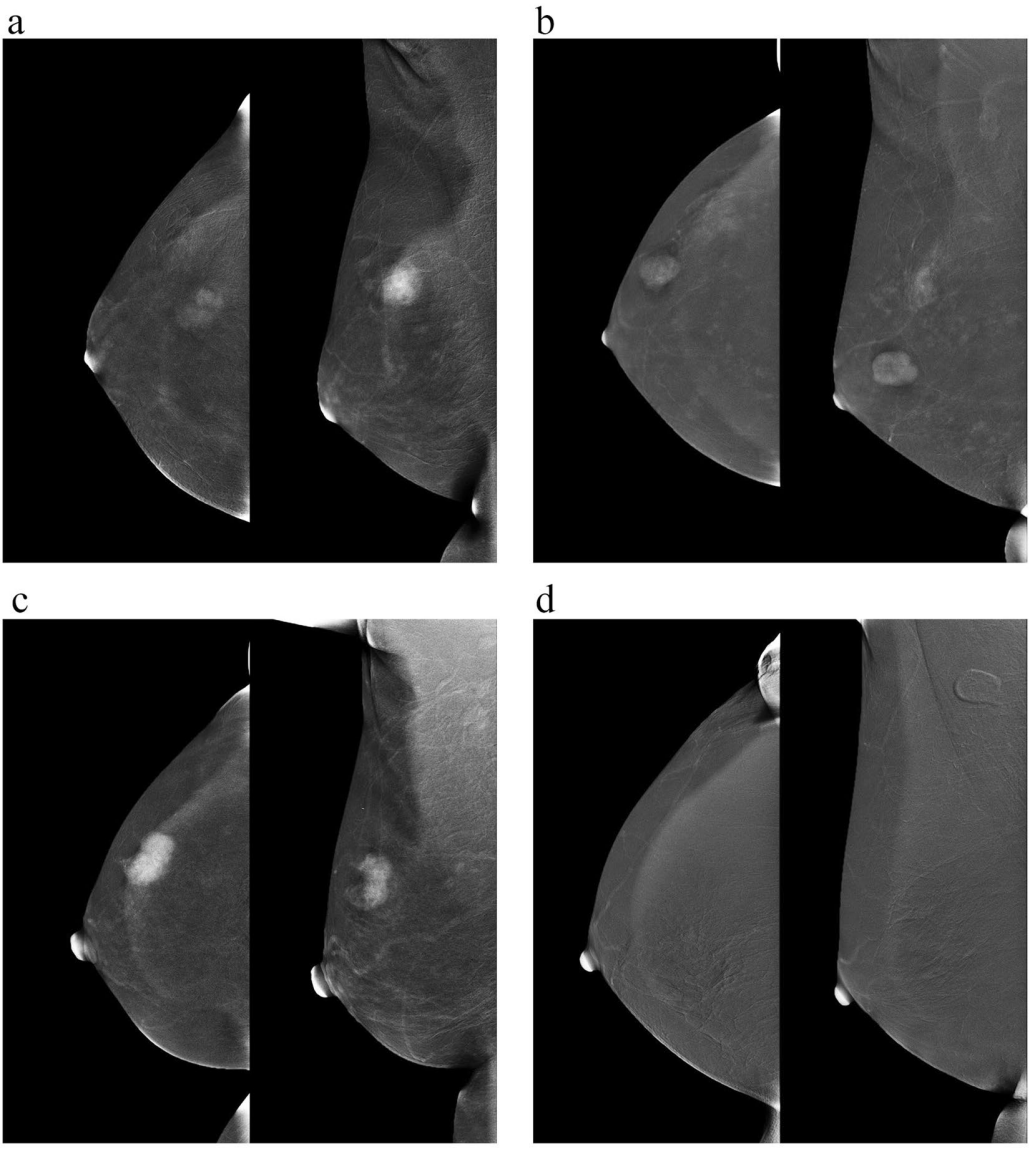
Four different enhancement patterns on CESM. According to RSD values, enhancement patterns were classified as **(a)** ascending pattern, **(b)** steady pattern, **(c)** descending pattern and **(d)** no enhancement.

On subtraction image, CNR or RSD value of a non-enhanced lesion was scored as zero. According to the changes of CNR values between the former and latter projections, enhancement patterns were classified as four types: ascending pattern, steady pattern, descending pattern and no enhancement (Fig. 2). Ascending pattern: the enhancement intensity showed a continuous rising state with the RSD > 10%. Steady pattern: the enhancement intensity displayed no noticeable difference between the two projections with the absolute value of RSD ≤ 10%. Descending pattern: the enhancement intensity in the latter position is weaker than the former one with RSD < −10%.

### DCE-MRI

MRI and CESM examinations were scheduled within one month. The breast MRI study was conducted on the 3.0 T imaging system (Discovery MR750W, GE Healthcare, Chalfont St. Giles, UK) with dedicated bilateral breast-surface coils. Patients were in prone position and T1- and fat-suppressed T2-weighted imaging and diffusion-weighted imaging were acquired before contrast. After intravenous gadolinium (0.2 ml/kg body weight, flow rate 2 ml/s), a dynamic series of fat-suppressed 3-dimensional T1-weighted images were collected. Based on the 5th edition of Breast Imaging Reporting and Data System (BI-RADS), TICs were categorized as persistent, plateau, or washout types.

### Histopathology

Pathological specimens were obtained by biopsy or surgical excision. Pathological classification and hormonal receptor status, including estrogen receptor (ER), progesterone receptor (PR) and human epidermal growth factor receptor-2 (HER-2), were recorded. According to immunohistochemistry (IHC), nuclear staining ≥10% was regarded as positive for ER and PR status. Expression of HER-2 protein was classified as 0, 1+, 2+, and 3+ by IHC. If a specimen was categorized as 2+, fluorescence *in situ* hybridization (FISH) was performed to test HER-2 gene. HER-2 positivity was defined as an IHC HER-2 score of 3+ or gene amplification by FISH. HER-2 negativity was defined as an IHC HER-2 score of 0 or 1+.

### Statistical analysis

Data analysis was conducted using SPSS Statistics (version 19.0, IBM, Armonk, NY) and MedCalc (version 15.6.1, MedCalc Software, Ostend, Belgium). For non-normal distribution parameters, descriptive statistics were expressed as the mean, median, minimum, maximum, and percentiles. Statistical inference was accomplished by Wilcoxon signed rank test for two relative groups, Mann-Whitney U test for two independent groups and Kruskal–Wallis test for more than two independent groups. Moreover, the Bonferroni method was utilized to adjust for multiple comparisons. The diagnostic performance of the examination was calculated compared to pathological findings. The cut-off values were based on the Youden’s J statistics and the diagnostic performances were assessed by analyzing the receiver operating characteristics (ROC) curves of subjects. Cohen’s kappa coefficient was calculated to measure the agreement between the CESM and MRI. In all tests, a *p*-values < 0.05 was deemed significant.

## Results

### Patients and lesions characteristics

For patients who had undergone CESM, we followed the clinical diagnostic and treatment process for more than 6 months, 145 lesions in 131 female patients were included for analysis. The mean patient age was 42 years (range, 18–77 years). In all, 102/145 (70.3%) were benign lesions and 43/145 (29.7%) were breast cancers. The benign lesions included fibroadenomas (n = 53), adenosis with or without fibrocystic changes (n = 33), intraductal papillomas (n = 8), atypical ductal hyperplasia (n = 3), usual ductal hyperplasia (n = 1), sclerosing adenosis (n = 1), inflammation (n = 1), duct ectasia (n = 1), and cyst (n = 1). There were 17 non-infiltrating cancers and 26 infiltrating cancers. These malignant lesions included ductal carcinoma *in situ* (n = 14), invasive ductal carcinoma (n = 23), solid papillary carcinoma associated with invasive ductal carcinoma (n = 2), invasive lobular carcinoma (n = 1), lobular carcinoma *in situ* (n = 1), mixed ductal carcinoma *in situ* associated and invasive lobular carcinoma (n = 1), and invasive adenocarcinoma (n = 1).

Of 43 breast cancers, we obtained ER and PR expression characteristics in 41 tumors, and HER-2 expression characteristics in 30 tumors. 30/41 (73.2%) were ER-positive cancers and 11/41 (26.8%) were ER-negative cancers. 24/41 (58.5%) were PR-positive cancers and 17/41 (41.5%) were PR-negative cancers. 9/30 (30.0%) were ER-positive cancers and 21/30 70.0%) were ER-negative cancers.

### CESM findings

LE images depicted 56 lesions, of which 28 were benign lesions and 28 were breast cancers. On subtraction images, 52.9% (54/102) of benign lesions and 97.7% (42/43) of cancers revealed enhancement and can be depicted. Subtraction images failed to show 49 lesions due to no distinguishable enhancement, including 48 benign lesions and 1 ductal carcinoma *in situ* (DICS). Of 54 enhanced benign lesions, 33 were diagnosed with fibroadenomas, 8 adenosis, and 6 intraductal papillomas, occupying 62.3% (33/53) of fibroadenomas, 24.2% (8/33) of adenosis, and 75.0% (6/8) of intraductal papillomas, separately. Other pathologic entities can also present as enhancement, but just account for a small percentage. In all 145 lesions, 49 only be found on subtraction images, but showed no visible abnormity on LE images, consisting of 15 malignancies and 34 benign lesions. However, 8 benign lesions and 1 DCIS only be detected on LE mammograms and can’t be observed on subtraction images. 40 lesions displayed no obvious abnormalities on both LE and subtraction mammograms, which were all proved to be benign. And 47 lesions (20 benign lesions and 27 breast cancer) can be detected by both examinations.

### Enhancement intensity on CESM

Table 1 provides the descriptive results of CNR values in different pathological diseases. In terms of the mean, median, maximum, and percentiles, enhancement intensity of breast cancers was significantly higher than that of benign lesions. Both CNR_1_ and CNR_2_ values of the three groups differed significantly (*p* < 0.001; *p* < 0.001). After the Bonferroni correction, the distribution of CNR_1_ values in benign lesions and non-infiltrating cancers (*p* = 0.001), and in benign lesions and infiltrating cancers (*p* < 0.001), was statistically significant, while the difference between non-infiltrating cancers and infiltrating cancers was not statistically significant (*p* = 1.000). The distribution of CNR_2_ values in benign lesions and non-infiltrating cancers (*p* < 0.001), and in benign lesions and infiltrating cancers (*p* < 0.001), was statistically significant, while the difference between non-infiltrating cancers and infiltrating cancers was not statistically significant (*p* = 1.000). For benign group, enhancement intensity in latter position was stronger than in former position (*p* = 0.028). Mean CNR_1_ was 2.7%, mean CNR_2_ was 3.0%, median CNR_1_ was 2.7%, and median CNR_2_ was 3.3%. By contrast, for breast cancers, enhancement intensity in latter position was weaker compared to former position (non-infiltrating cancers, *p* = 0.301; infiltrating cancers, *p* = 0.006). In non-infiltrating group, mean CNR_1_ was 6.5%, mean CNR_2_ was 6.2%, median CNR_1_ was 6.8%, and median CNR_2_ was 5.6%. In infiltrating group, mean CNR_1_ was 7.6%, mean CNR_2_ was 6.4%, median CNR_1_ was 6.8%, and median CNR_2_ was 5.6%.

**Table 1:** Detailed descriptive results of CNR values of different pathological diseases.

CNR Values		Benign Lesions (n = 102)		Non-infiltrating Cancers (n = 17)		Infiltrating Cancers (n = 26)	
		Former Position	Latter Position	Former Position	Latter Position	Former Position	Latter Position
Mean		2.7%	3.0%	6.5%	6.2%	7.6%	6.4%
Median		2.7%	3.3%	6.8%	5.6%	6.8%	5.6%
Minimum		0	0	0	0	1.8%	0.6%
Maximum		10.1%	13.2%	11.8%	10.4%	27.2%	26.0%
Percentiles	25	0.0%	0.0%	5.0%	4.3%	4.9%	4.3%
	50	2.7%	3.3%	6.8%	5.6%	6.8%	5.6%
	75	4.8%	5.2%	8.1%	8.5%	9.2%	7.5%
*p-*value		0.028		0.301		0.006	

The sensitivities were 81.4% for CNR_1_ and 93.0% for CNR_2_. The specificities were 75.5% for CNR_1_ and 52.9% for CNR_2_. The area under the ROC curves (AUCs) were 0.843 (95% confidence interval [CI]: 0.773–0.898; *p* < 0.0001) for CNR_1_, and 0.755 (95% CI: 0.677–0.822; *p* < 0.0001) for CNR_2_. The AUC was significantly higher for CNR_1_ than it was for CNR_2_ (*p* = 0.0008).

We used CNR_1_ to value enhancement intensity of cancers with different hormonal receptor status (Table 2). Enhancement intensity of ER positive group was weaker than ER negative group (*p* = 0.037). For ER positive and negative cancers, mean CNR_1_ was 6.4% vs. 9.7%, and median CNR_1_ was 5.9% vs. 7.7%. Enhancement intensity of PR positive group was weaker than PR negative group (*p* = 0.508). For PR positive and negative cancers, mean CNR_1_ was 6.6% vs. 8.1%, and median CNR_1_ was 6.2% vs. 7.0%. Enhancement intensity of HER-2 positive group was stronger than HER-2 negative group (*p* = 0.283). For HER-2 positive and negative cancers, mean CNR_1_ was 7.6% vs. 6.4%, and median CNR_1_ was 7.2% vs. 6.7%.

**Table 2:** Detailed descriptive results of CNR_1_ values of breast cancers with different hormonal receptor status.

CNR_1_ Values		ER Status (n = 41)		PR Status (n = 41)		HER-2 Status (n = 30)	
		Positive (n = 30)	Negative (n = 11)	Positive (n = 24)	Negative (n = 17)	Positive (n = 9)	Negative (n = 21)
Mean		6.4%	9.7%	6.6%	8.1%	7.6%	6.4%
Median		5.9%	7.7%	6.2%	7.0%	7.2%	6.7%
Minimum		0	4.4%	0.0%	3.4%	5.4%	0.0%
Maximum		11.8%	27.2%	11.8%	27.2%	10.5%	11.8%
Percentiles	25	4.8%	6.9%	4.8%	5.7%	6.3%	4.6%
	50	5.9%	7.7%	6.2%	7.0%	7.2%	6.7%
	75	8.1%	10.2%	8.9%	8.5%	9.1%	8.2%
*p-*value		0.037		0.508		0.283	

### Enhancement patterns on CESM and MRI

Detailed descriptive results of RSD values are listed in Table 3. Mean RSD of benign group was 11.0% and median value was 0, while mean RSD of malignancies was −9.7% and median value was −11.9%. The differences of RSD values between benign and malignant lesions were significant (*p* < = 0.001). Enhancement patterns on CESM in different pathological diseases are provides in Table 4. As for benign group, ascending pattern accounted for 25.5% (26/102), steady pattern 16.7% (17/102), descending pattern 10.8% (11/102), and non-enhanced group 47.1% (48/102). The proportions of enhancement patterns in malignancies were 18.6% (8/43) for ascending pattern, 27.9% (12/43) for steady pattern, 51.2% (22/43) for descending pattern, and 2.3% (1/43) for non-enhanced type. The distribution of enhancement patterns in benign and malignant groups was statistically different (*p* < 0.001).

**Table 3:** Detailed descriptive results of RSD values of different pathological diseases.

RSD values		Benign (n = 102)	Malignant (n = 43)	*p-*value
Mean		11.0%	−9.7%	<0.001
Median		0	−11.9%	
Minimum		−33.1%	−70.8%	
Maximum		215.1%	48.5%	
Percentiles	25	0	−24.4%	
	50	0	−11.9%	
	75	11.4%	6.0%

**Table 4:** Enhancement patterns on CESM in different pathological diseases. Percentages are in parentheses.

Enhancement patterns	Benign (n = 102)	Malignant (n = 43)	*p*-value
No enhancement	48 (47.1%)	1 (2.3%)	<0.001
Ascending pattern	26 (25.5%)	8 (18.6%)	
Steady pattern	17 (16.7%)	12 (27.9%)	
Descending pattern	11 (10.8%)	22 (51.2%)	

28 patients with 28 lesions performed both CESM and DCE-MRI examinations. The results of TICs on DCE-MRI are listed as follows: in 12 benign lesions, 33.3% (4/12) without enhancement, 25.0% (3/12) with persistent types, 25.0% (3/12) with plateau types, and 16.7% (2/12) with washout types; in 16 malignant lesions, 12.5% (2/16) with persistent types, 50.0% (8/16) with plateau types, and 37.5% (6/16) with washout types. The distribution of TUCs in benign and malignant groups was statistically different (*p* = 0.046).

Comparing enhancement patterns on CESM and TICs on DCE-MRI, the results demonstrated that 2/28 (7.1%) lesions with a concordant ascending pattern (or persistent type), 5/28 (17.8%) lesions with a concordant steady pattern (or plateau type), 7/28 (25.0%) lesions with a concordant descending pattern (or washout type), 4/28 (14.3%) lesions with no enhancement, and 10/28 (35.7%) lesions with inconsistent enhancement patterns. The coincidence rate of two modalities was 64.2%. Cohen’s kappa showed moderate agreement (k = 0.515) between CESM and MRI.

For 96 enhanced lesions, an average time gap between the former and latter projections of suspicious breast was 104 seconds (range, 54–227 seconds). Images of CC position were projected prior to MLO position in a large portion of cases (79 lesions), and MLO views were projected prior to CC views in 17 lesions. Among 42 enhanced cancers, 37 lesions projected CC views first and 5 projected MLO views first, respectively, accounting for 21.6% (8/37) and 0 in ascending pattern, 21.6% (8/37) and 80.0% (4/5) in steady pattern, 56.8% (21/37) and 20.0% (1/5) in descending pattern. By contrast, 42 benign lesions projected CC views first and 12 projected MLO views first, respectively, occupying 47.6% (20/42) and 50.0% (6/12) in ascending pattern, 35.7% (15/42) and 16.7% (2/12) in steady pattern, 16.7% (7/42) and 33.3% (4/12) in descending pattern. There was no statistically significant difference in the distribution of enhancement patterns between CC prior to MLO and MLO prior to CC conditions in benign group (*p* = 0.304), while the difference was significant in malignancies (*p* = 0.027).

## Discussion

Based on the angiogenesis, breast lesions can be clearly demonstrated on CESM by eliminating the overlapping of breast fibroglandular tissues^4–6^. CESM is superior to conventional mammography and improves breast cancer detection^10^. In this study, CESM demonstrated 97.6% (42/43) of malignancies, while LE only revealed 65.1% (28/43) of cancers, indicating a higher cancer detection rate on CESM. However, we observed that 51.5% (51/102) of benign lesions revealed enhancement on CESM, which means that it is not proper to diagnose the lesions depending on whether it is enhanced or not. In order to explore enhancement characteristics between benign and malignant lesions and improve diagnostic specificity, we conducted this study. We quantitatively assessed enhancement intensity of breast lesions with different pathological types and hormonal receptor status on CESM and evaluated the consistency of enhancement patterns between CESM and DCE-MRI.

For enhancement intensity, this preliminary research suggested that enhancement intensity of malignancies was usually stronger than that of benign lesions (Table 1), which was consistent with other researchers’ findings^6,11–14,17^. Lewin *et al*.^6^ concluded that 84.6% (11/13) of invasive cancers enhanced strongly, while all benign lesions (n = 12) were presented as no or weak enhancement qualitatively. Luczynska *et al*.^14^ observed medium or strong enhancement among 83% (106/127) invasive carcinomas while no enhancement or weak enhancement among 71% (58/82) benign lesions. Using a quantitative program, Deng *et al*.^13^ reported that the mean pixel value of malignant lesions was 476.05 (versus 85.79 for benign lesions).

Rudnicki *et al*.^12^ and our researches took a quantitative method to evaluate enhancement intensity of lesions on the both two projections (CC and MLO views). Rudnicki *et al*. adopted the percentage signal difference between enhancing lesion and background (%RS) to value the intensity of lesions, which was similar to CNR index in this research. The only difference between %RS and CNR was that Rudnicki *et al*. measured mean signals of lesions, whereas we measured maximum signals, which took into consideration that the heterogeneity of lesions may had effect on mean signals and caused unstable results. Both we found that mean %RS and CNR values were correlated with pathological classification. Comparison of three different pathological classifications indicated that infiltrating cancers showed the highest values, while benign lesions the lowest.

In CESM protocols, Rudnicki *et al*. performed CC views prior to MLO views. While the sequence of CC or MLO position was not fixed in this study, CNR_1_ was defined to present enhancement intensity in the former projection and CNR_2_ to present the latter projection. According to Rudnicki’s data, the AUCs were 0.725 in the former projection and 0.713 in the latter projection. They concluded that the difference between two views was not significant and two views had similar diagnostic accuracy, which probably because the time interval between two projections was not long enough. But we came up with different results. We calculated an AUC of 0.843 in the former projection and 0.755 in the latter projection and found a significant difference of enhancement intensity between two views, indicating that the former projections of contrast-enhanced images seemed to play a more important role in the diagnosis of breast lesions compared with the latter projective views.

Marino *et al*.^18^ had found that CESM and radiomics analysis had differential value in hormonal receptor status with accuracy of 78.4% to 100%. This study analysed CNR differences between receptor positive and negative lesions and concluded that enhancement intensity was related to hormonal receptor status. Enhancement intensity of ER or PR positive group was weaker than negative group and HER-2 positive group was stronger than negative group, of which the signal discrepancy between ER positive and negative lesions was statistically significant. It suggested that CESM may contributed to differentiate hormonal receptor status. Due to insufficient sample size, the results needed further confirmation.

As indicated in Table 1, CNR_2_ was lower than CNR_1_ in malignant group, while CNR_2_ was higher than CNR_1_ in benign group, which revealed a significant discrepancy of enhancement intensity between the former and latter projections. According to this, we further investigated enhancement patterns of breast lesions. The classification of enhancement patterns on CESM was similar to the TIC on breast DCE-MRI. On MRI, Kuhl *et al*.^19^ showed enhancement types among 101 malignant lesions as follows: persistent enhancement, 8.9%; plateau enhancement, 33.6%; and washout enhancement 57.4%. For 165 benign lesions, results were as follows: persistent enhancement, 83.0%; plateau enhancement, 11.5%; and washout enhancement 5.5%. On CESM, previous studies^4,13,15^ had described enhancement patterns of breast lesions. Both Jong *et al*.^15^ and Dromain *et al*.^4^ took consecutive exposures in a small number of patients. Jong revealed that one of 10 (10.0%) malignancies and 3 of 12 (25.0%) benign lesions with increasing enhancement, 4 of 10 (40.0%) and one of 12 (8.3%) with plateau enhancement, 3 of 10 (30.0%) and one of 12 (8.3%) with washout enhancement, 2 of 10 (20.0%) and 7 of 12 (58.3%) without enhancement. Excluding one case of interference due to motion artifacts, Dromain showed that 7 of 19 (36.8%) breast cancers with increasing enhancement, 4 of 19 (21.1%) with plateau enhancement, 4 of 19 (21.1%) with washout enhancement, and 4 of 19 (21.1%) with no enhancement. Deng evaluated the relative enhancements between early and late phases and reported the proportions of enhancement patterns in 44 benign lesions and 106 breast cancers, respectively, were 15.9% and 17.9% for elevated pattern, 2.3% and 12.3% for steady pattern, 9.1% and 62.3% for depressed pattern, and 72.7% and 7.5% for non-enhanced pattern. As to our findings, in 102 benign lesions and 43 malignancies, separately, ascending type accounted for 25.5% and 18.6%, steady type accounted for 16.7% and 27.9%, descending type accounted for 10.8% and 51.2%, and non-enhanced lesions accounted for 47.1% and 2.3%. No matter on MRI or CESM, malignant lesions had a tendency to demonstrate washout enhancement (depressed or descending patterns) and the majority of benign lesions showed non-enhanced or persistent enhancement (elevated or ascending patterns), which may be of great help to differentiate benign and malignant lesions.

Previously, Froeling *et al*.^20^ had found a correlation of contrast agent kinetics between contrast-enhanced digital breast tomosynthesis and MRI, showing a moderate agreement (k = 0.438). Inspired by this, we compared the enhancement patterns between CESM and MRI and achieved a coincidence rate of 64.2% and moderate agreement (k = 0.515), which demonstrated that there was a certain consistency of enhancement patterns between two modalities.

So far, the order of image acquisition (CC prior to MLO or MLO prior to CC) on CESM was controversial^21^. In published papers, some exposed MLO views first^11,16,22^, while others performed CC views first^12,13^. In our CESM protocols, the sequence of CC or MLO position was not fixed. The distribution of enhancement patterns was not statistically different between CC prior to MLO and MLO prior to CC conditions in benign lesions, while the difference was significant in malignant lesions. So, it remained uncertain whether the projective order have effect on enhancement patterns.

**Figure 3:**
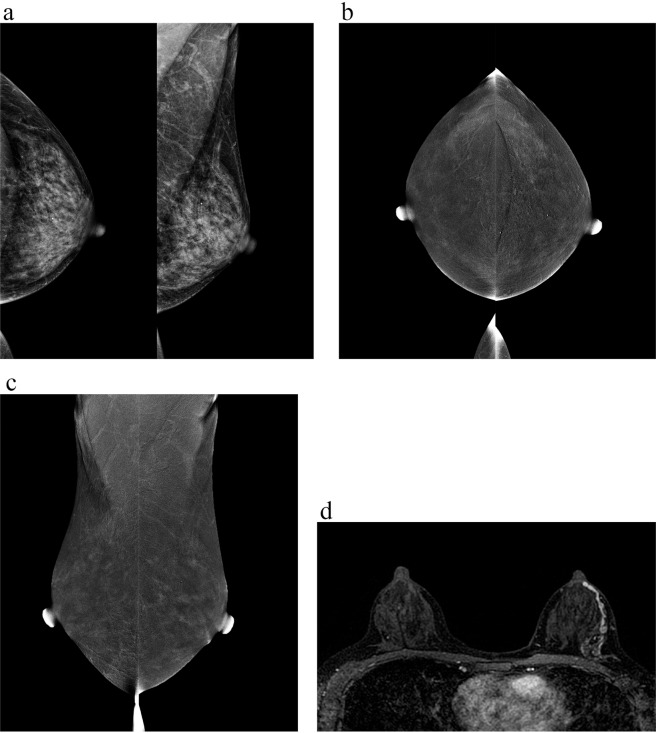
A 47-year-old woman with one ductal carcinoma *in situ* in the left breast at 3 o’clock. (**a**) Low-energy images in CC and MLO views show scattered microcalcifications in the left breast. (**b)** Subtraction images in CC views and **(c)** MLO views display symmetrical moderate background parenchyma enhancement in bilateral breasts and no distinct enhancing areas stronger than background enhancement are detected. **(d)** Contrast-enhanced MRI depicted non-mass enhancement with linear distribution.

One DCIS with no distinguishable enhancement is displayed in Fig. 3. Scattered microcalcifications in the left breast were detected on LE images. On subtractions images, there was a symmetrical moderate background parenchyma enhancement in bilateral breasts and no distinct enhancing area stronger than background enhancement was found. Contrast-enhanced MRI depicted non-mass enhancement with linear distribution in the left breast. The biopsy result of microcalcifications located in the left breast at 3 o’clock gave a diagnosis of atypical hyperplasia. Then the left mastectomy was performed and frozen section examination suggested multiple atypical hyperplasia in the left breast, and immunohistochemical analysis clarified DCIS. Accordingly, a comprehensive analysis combined with other examination results is necessary. The authors believe that not a single standard is all-powerful, and only make the most of complementary advantages of multiple modalities (such as mammography, ultrasound, MRI) can maximize the benefits to patients.

A major limitation of the current study was that sample size was insufficient, especially the malignant cases, and the diagnostic value of quantitative methods needs to be confirmed in a large-scale clinical experience. In addition, the number of cases in which MLO projected first was too small to determine whether the projection sequence affected enhancement pattern.

Finally, we come to the conclusion that enhancement intensity and patterns on CESM have relevance to pathological types of breast lesions and have good performances in the diagnosis of breast cancers. The enhancement intensity of malignancies was usually stronger than that of benign lesions. Besides, enhancement intensity may contribute to differentiate hormonal receptor status. In terms of enhancement patterns, a large proportion of malignant lesions have a tendency to demonstrate descending patterns and the majority of benign lesions show non-enhanced or ascending patterns. Moreover, CESM examinations show a moderate agreement on enhancement patterns with DCE-MRI. In conclusion, quantitative analysis of enhancement characteristics is feasible to the clinical practice on CESM.

## Data Availability

The datasets generated during and/or analysed during the current study are available from the corresponding author on reasonable request.
